# Advantages of Additive Manufacturing for Biomedical Applications of Polyhydroxyalkanoates

**DOI:** 10.3390/bioengineering8020029

**Published:** 2021-02-23

**Authors:** Alberto Giubilini, Federica Bondioli, Massimo Messori, Gustav Nyström, Gilberto Siqueira

**Affiliations:** 1 Department of Engineering and Architecture, University of Parma, 43124 Parma, Italy; alberto.giubilini@studenti.unipr.it; 2 Department of Applied Science and Technology, Politecnico di Torino, 10129 Torino, Italy; federica.bondioli@polito.it; 3 Department of Engineering “Enzo Ferrari”, University of Modena and Reggio Emilia, 41125 Modena, Italy; massimo.messori@unimore.it; 4 Cellulose & Wood Materials Laboratory, Empa—Swiss Federal Laboratories for Materials Science and Technology, 8600 Dübendorf, Switzerland; gustav.nystroem@empa.ch; 5 Department of Health Sciences and Technology, ETH Zürich, 8092 Zürich, Switzerland

**Keywords:** polyhydroxyalkanoates, scaffolds, biomedicine, additive manufacturing, 3D printing, drug delivery, vessel stenting, tissue engineering

## Abstract

In recent years, biopolymers have been attracting the attention of researchers and specialists from different fields, including biotechnology, material science, engineering, and medicine. The reason is the possibility of combining sustainability with scientific and technological progress. This is an extremely broad research topic, and a distinction has to be made among different classes and types of biopolymers. Polyhydroxyalkanoate (PHA) is a particular family of polyesters, synthetized by microorganisms under unbalanced growth conditions, making them both bio-based and biodegradable polymers with a thermoplastic behavior. Recently, PHAs were used more intensively in biomedical applications because of their tunable mechanical properties, cytocompatibility, adhesion for cells, and controllable biodegradability. Similarly, the 3D-printing technologies show increasing potential in this particular field of application, due to their advantages in tailor-made design, rapid prototyping, and manufacturing of complex structures. In this review, first, the synthesis and the production of PHAs are described, and different production techniques of medical implants are compared. Then, an overview is given on the most recent and relevant medical applications of PHA for drug delivery, vessel stenting, and tissue engineering. A special focus is reserved for the innovations brought by the introduction of additive manufacturing in this field, as compared to the traditional techniques. All of these advances are expected to have important scientific and commercial applications in the near future.

## 1. Introduction

The term “biopolymer” is nowadays very common and widely spread in different fields of application. However, it is sometimes improperly used, due to the fact that there is not a brief and comprehensive definition of this word. To clarify the meaning of “biopolymer”, it is important to define the concepts of “bio-based” and “biodegradable”, and if the former is strictly connected with the origin of the material, at the opposite, the latter is related to its end-of-life.

A material can be defined as bio-based if it derives in whole or in part from biomass resources, i.e., organic materials that are renewable [1].

A material can be properly defined as biodegradable if it can be used as a carbon source by microorganisms and converted safely into CO_2_, biomass and water [2]. Besides, if the material undergoes a biodegradation and a physical disintegration level of at least 90%, in less than six months, then it can also be defined as “compostable” [3].

Hence, the family of biopolymers can be divided into three main groups:

1. Biopolymers coming from renewable resources but not being biodegradable, e.g., bio-based polyethylene terephthalate (bio-PET), bio-based polypropylene (bio-PP), and bio-based polyethylene (bio-PE);
2. Biopolymers coming from not-renewable resources but being biodegradable, e.g., polybutylene adipate terephthalate (PBAT);
3. Biopolymers coming from renewable resources and being biodegradable, e.g., polyhydroxyalkanoate (PHA), poly(lactic acid) (PLA), and polybutylene succinate (PBS).

**Figure 1:**
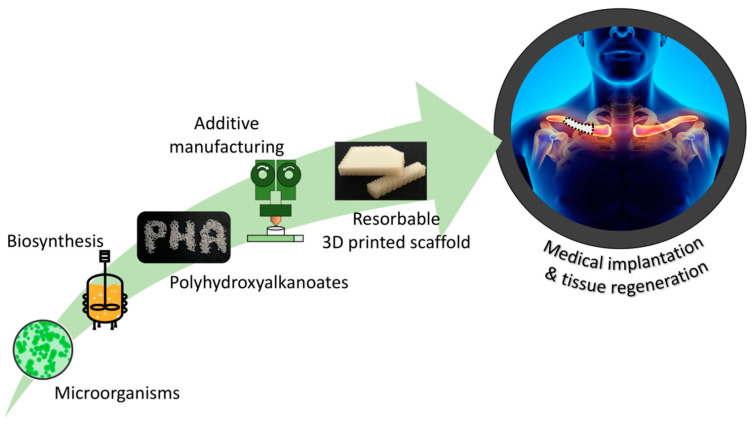
Schematic representation of the production, technological transformation, and biomedical applications of polyhydroxyalkanoate (PHA)-based devices.

In this review, the PHA family is taken into consideration, and particular interest is reserved to its application in the biomedical field. Since the beginning of the twenty-first century, an increasing number of scientific studies and clinical trials have been published about PHA medical devices for different final applications, such as tissue engineering, drug delivery, or as vascular stents [4]. Therefore, first this review is aimed to present and discuss the results obtained with PHA and traditional techniques, like solvent casting, phase separation, salt leaching, or electrospinning. Furthermore, a great importance is given to the introduction of additive manufacturing in this research field, and particularly to the innovations and advantages introduced by 3D printing, which allowed us to overcome some of the greatest limitations of traditional approaches. For example, thanks to additive manufacturing, it was possible to obtain a finer control over the porosity, a true development of the devices in all three dimensions, and even the reproduction of complex structures, which are able to mimic natural tissues and which are highly tailored to the physical requirements of each patient [5,6]. Finally, this review is concluded with a discussion, in the authors’ opinion, of the most likely future biomedical perspectives for this promising class of biopolymer, and of the new targets that can be achieved, thanks to 3D printing, in a new way of considering medicine, with a high customization of medical care. In Figure 1 shows a schematic representation of the overall topic and structure of this review work.

The methodology carried out for the analysis of the literature started with searching published reviews on two of the most widespread databases, i.e., Scopus and ScienceDirect. Keywords selected for the literature search included PHA, additive manufacturing, biomedical application, biopolymer medical device, and PHA biosynthesis. These reviews were scanned, all parts related to PHA were highlighted, and the cited original research articles were acquired. After that, all references’ abstracts were examined, and a first category clustering was performed according to this filtering system: (1) PHA production; (2) traditional PHA medical devices (solvent casting, salt leaching, thermally induced phase separation, non-solvent induced phase separation, emulsification, and electrospinning); (3) innovative PHA medical devices (Direct Ink Writing, Fused Deposition Modeling, Selective Laser Sintering, and Computer Aided Wet-Spinning). Afterwards, a second classification was implemented, to order all the references in accordance with the final medical application: (1) drug delivery; (2) vessel stenting; (3) bone tissue engineering, and (4) cartilage tissue engineering. Eventually, a combination of the two former groups was completed, and this synthesis was used as starting point for the manuscript development.

## 2. PHA: Biosynthesis and Properties

Due to the global awareness of the environmental impact of fossil-based polymers [7], the main goal of plastic industry is nowadays to tackle plastic pollution and its sociopolitical and economic challenges by developing new materials that can combine the advantages of traditional plastics with a sustainable production and disposal. In this research field, biopolymers play a central role due to their great benefits, such as carbon footprint reduction, saving of fossil resources and landfill decrease [8]. 

**Figure 2:**
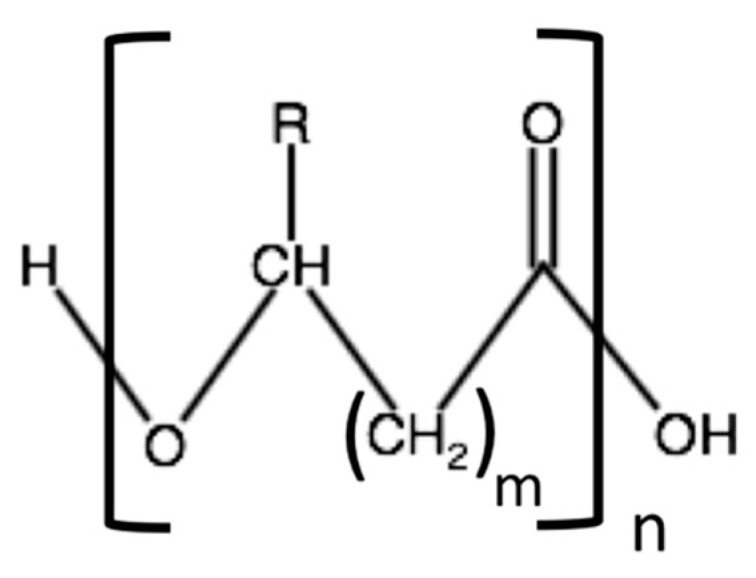
General chemical structure of polyhydroxyalkanoates (PHAs); “m” varies from 1 to 4 and “n” ranges from 100 to 30,000; R denotes a hydrogen atom or an alkyl side chain [12].

PHA is a large family of thermoplastic aliphatic polyesters mainly produced by prokaryotic organisms, such as bacteria, most prevalently Gram-negative [9], and archaea under conditions of nutrient depletion and in the presence of an excess of carbon source [10]. It is noteworthy to consider that, although only at a preliminary scientific research level, the production of PHAs from plants was achieved [11]. The general structure of PHAs is reported in Figure 2, where m can be equal or greater than one and R can be a hydrogen atom or an alkyl substituent, depending on the type of PHA [12]. Maurice Lemoigne, a French microbiologist, was the first researcher who identified the synthesis of PHAs from bacteria in 1926 by using a culture of *Bacillus megaterium* to isolate poly(3-hydroxybutyrate) (PHB) [13].

As several biopolymers belong to the PHA family, their classification is important, and they can be sorted depending on their chain-length monomeric composition, according to the number of carbon atoms per monomer, and here a great importance is played by the composition of the monomer side chain R [14]:

- Short-chain-length PHA (scl-PHA) has three to five carbon atoms;
- Medium-chain-length PHA (mcl-PHA) has 6 to 14 carbon atoms;
- Long-chain-length PHA (lcl-PHA) has more than 14 carbon atoms.

Generally, scl-PHAs, containing mainly 3-hydroxybutyrate (3HB) or 3-hydroxyvalerate (3HV) units, have a higher degree of crystallinity, a higher glass transition temperature, and a higher molecular mass compared to mcl-PHAs [15,16,17], containing 3-hydroxyhexanoate (3HH), 3-hydroxyoctanoate (3HO), 3-hydroxydecanoate (3HD), or 3-hydroxydodecanoate (3HHD) monomers.

**Figure 3:**
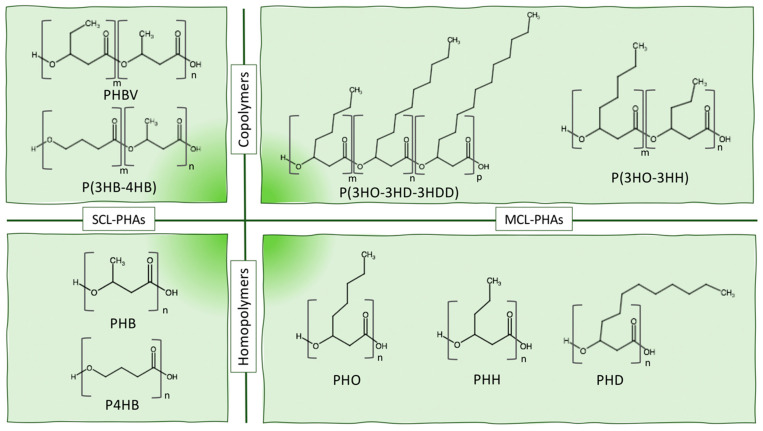
PHAs classification depending on the chain length and the chemical structure of the monomers. PHBV—poly(3-hydroxybutyrate-*co*-3-hydroxyvalerate); P(3HB-4HB)—poly(3-hydroxybutyrate-*co*-4-hydroxybutyrate); PHB—poly(3-hydroxybutyrate); P4HB—poly(4-hydroxybutyrate); P(3HO-3HD-3HDD)—poly(3-hydroxyoctanoate-*co*-3-hydroxydecanoate-*co*-3-hydroxydodecanoate); P(3HO-3HH)—poly(3-hydroxyoctanoate-*co*-3-hydroxyhexanoate); PHO—poly(3-hydroxyoctanoate); PHH—poly(3-hydroxyhexanoate); PHD—poly(3-hydroxydecanoate).

Another possible distinction can be made between homopolymer, of which the most famous and widespread example is PHB, and copolymers, such as poly(3-hydroxybutyrate-*co*-3-hydroxyvalerate) (PHBV), poly(3-hydroxybutyrate-*co*-4-hydroxybutyrate) (P3HB-4HB), or poly(3-hydroxybutyrate-*co*-3-hydroxyhexanoate) (PHBH). In this latter case, also the monomer arrangement can define a further method of classification. In fact, the difference between block copolymers and random copolymers is due to the ordered succession of similar monomers, unlike a random distribution, distinctive of the second type of copolymers [18]. The physical blending or the chemical copolymerization allow us to obtain a final material with tuned properties, which directly depend on the structures of the single-constituent monomers [10]. For example, PHB has a high crystallinity and brittleness, which can be reduced by introducing a new monomer unit, such as 3HV or 3HH [19]. The molar composition ratio of the copolymers is a key factor to tune the final properties, such as elongation at break and degree of crystallinity, which increase with the increase of 3HV [20] or 3HH [21] molar content in the structure. Figure 3 shows a schematic representation and categorization of the PHA family according to the chain-length and the composition of the structural units.

**Figure 4:**
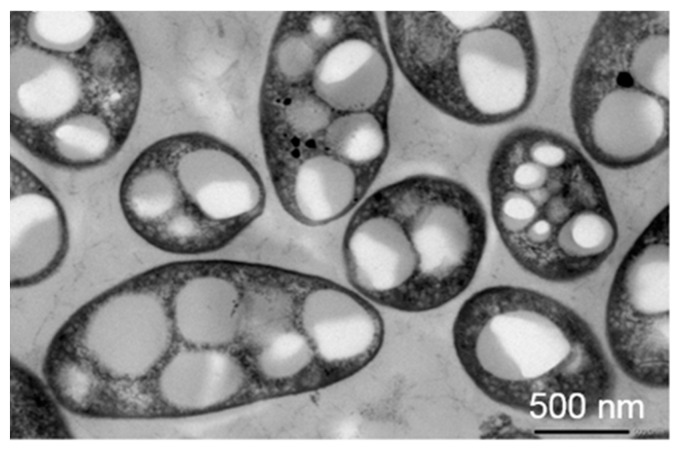
TEM image of *Rhodovulum visakhapatnamense* accumulating intracellular PHA granules, appearing as whitish and bright areas (adapted from Reference [25]).

As already reported, PHAs are bio-based polymers, whose origin derives from bacterial and archaeal fermentation. Some microorganisms, when they are subjected to an environmental stress, such as a depletion of essential nutrients, can start a conversion of the carbon sources in hydroxyalkanoate units, such as carbon and energy reserve, which are further polymerized into PHA granules through a biosynthetic pathway and stored in the bacterial cell cytoplasm [22]. The average size of the PHA granules is approximately 0.2–0.5 μm [23,24]. In Figure 4, a transmission electron micrograph of *Rhodovulum visakhapatnamense* cells containing PHA granules is reported.

**Figure 5:**
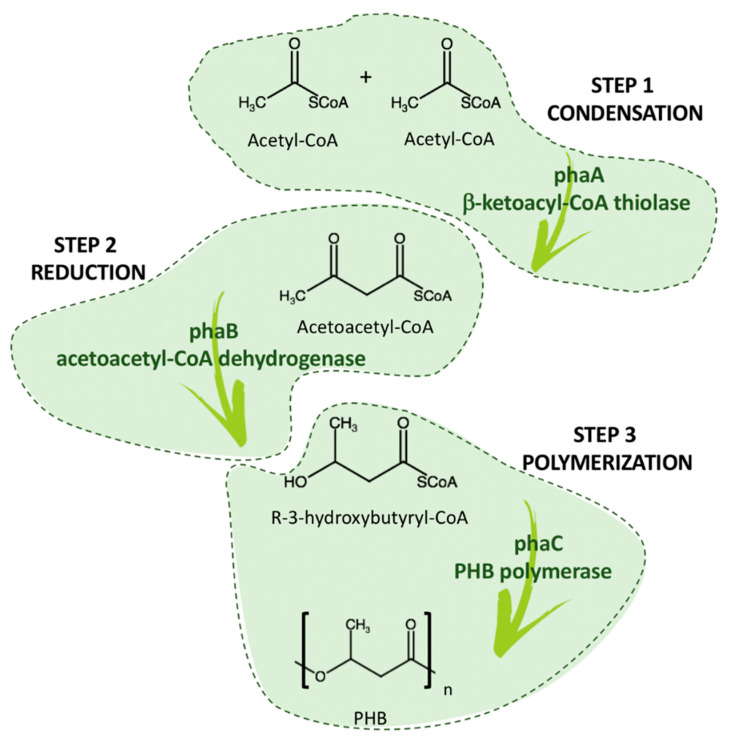
Biosynthetic pathway of poly(3-hydroxybutyrate) production within the bacterial cytoplasm. PHB is synthesized by the successive action of three enzymes: β-ketoacyl-CoA thiolase (phbA), acetoacetyl-CoA dehydrogenase (phbB), and PHB polymerase (phbC) in a three-step pathway.

The biosynthetic pathway of PHB consists of three enzymatic reactions catalyzed by three different enzymes: phbA, phbB, and phbC. The first reaction is a condensation of two acetyl coenzyme A (acetyl-CoA) molecules into acetoacetyl-CoA by β-ketoacyl-CoA thiolase (encoded by phbA). The second reaction is the reduction of acetoacetyl-CoA to (R)-3-hydroxybutyryl-CoA by an NADPH-dependent acetoacetyl-CoA dehydrogenase (encoded by phbB). Lastly, the (R)-3-hydroxybutyryl-CoA monomers are polymerized into PHB by PHB polymerase (encoded by phbC) [26,27]. The scheme in Figure 5 synthesizes the fundamental enzymatic biosynthetic pathway.

Nowadays, the number of bacteria that is able to produce PHA is remarkable, i.e., more than eighty different genera [22]. The most commonly used bacteria species able to produce PHAs belong to the genera of *Alcaligenes*, *Azotobacter*, *Bacillus*, *Cupriavidus*, *Chromobacterium*, *Delftia*, *Pseudomonas*, *Ralstonia*, and *Staphylococcus* [28]. Different microorganisms own different polymerase enzymes, and this leads to the fact that every single microorganism is capable of producing small differences in the final biopolymer [29]. For example, *Ralstonia* bacteria have a particular polymerase enzyme that prioritizes the synthesis of scl-PHA [30]; on the opposite, *Pseudomonas* bacteria produce mcl-PHA [31]. Moreover, the PHA production yield can vary significantly from 0.25 g/L, using, for example, terephthalic acid as carbon source for *Pseudomonas putida* GO16, to 51.2 g/L, using commercial glycerol as carbon source for *Cupriavidus necator* DSM 545 [10].

Carbon is at the basis of organic chemistry and the fundamental element for all biomasses. There are different possible carbon sources that can be used to feed the microorganisms during PHA production and they can be classified in three different substrate groups: carbohydrates (e.g., sucrose, lactose, starch, or lignocellulose) [32,33,34], triacylglycerols (e.g., animal fats or plant oils) [35,36], and hydrocarbons. The last group is not economically significant since only few species of bacteria are capable to synthetize PHAs from this source and the process tends to have a low efficiency [37]. Apart from the carbon, other chemical compounds are required such as nitrogen sources, and some of the most used are (NH_4_)_2_SO_4_, NH_4_Cl, or NH_4_NO_3_ [22]. Variation in carbon to nitrogen ratios led to a different amount of PHA concentration in bacterial cells [38], and most of the studies showed that limiting nitrogen concentration while increasing carbon substrates had a positive effect on the PHA production rate [39,40]. Since the biosynthesis process ends with the storage of PHA granules into the cell cytoplasm, a further crucial step is required, the extraction of the PHAs granules from the bacterial cell. The approaches for biopolymer recovery can be different, and they are here synthesized:

- Solvent dissolution: The extraction is performed on pretreated cells, where PHA granules were made accessible by rupture of the cell membrane, and halogenated solvents are then used to dissolve the granules and then precipitate them in a non-solvent solution [41]. The biggest limitation of this method is the need of a high amount of harmful solvents, which hinders the environmental benefits of PHA biosynthesis [42]. In order to overcome this drawback, the use of non-halogenated solvents or supercritical CO_2_ are being investigated as alternatives [43].
- Enzymatic digestion: This method consists of a digestion of the cell membrane by action of enzymes, followed by filtration, floatation, or centrifugation recovery of the PHA granules [44].
- Chemical digestion: The procedure consists, as in the previous procedure, of the digestion of the cell membrane by the chemical action of sodium hypochlorite at high pH values, which makes most of the cellular components soluble in water, due to oxidation, and therefore easily removable [45].
- Mechanical disruption: The microbial cells are mechanically disintegrated by high-pressure homogenization or ultrasonication, thus making PHA granules recuperable [46].
- Osmophilic disruption: The rupture of the cell is caused by the high internal pressure in hypotonic media due to osmotic absorption, which causes the release of the intracellular content [47].
- Biological extraction: This ecological procedure consists of the use of insects, such as the mealworm, that can be fed on lyophilized cells of *Cupriavidus necator*, with intracellular PHB granules. Once the feeding is complete, PHB can be extracted from the fecal pellets of the black soldier fly larvae [48].

The choice of the most suitable recovery method depends on several factors such as the microbial strain, the type of PHA and the required purity grade of the final product. Specifically, the purity of the polymer has a critical importance for biomedical applications. In fact, biological active contaminants, such as endotoxins, can cause undesired immunological responses. For example, the US Food and Drug Administration (FDA) regulations limited the endotoxin content of medical devices to 20 USP endotoxin units per device, and to 2.15 in case of devices associated with the cerebrospinal fluid [49]. So far, different approaches have been suggested, but there is still room for improvement and innovation on this particular aspect. Burniol-Figols et al. evaluated an innovative PHA purification through dilute aqueous ammonia digestion (purity 86 ± 0.8%), and they compared it with reference processes, such as dissolution in chloroform and precipitation in methanol (purity 99 ± 0.2%), or also acid-mediated digestion with H_2_SO_4_, followed by a treatment with NaOCl and subsequent washing with water and centrifugation (purity 98 ± 2.6%) [50]. Moreover, more environmentally friendly purification processes were proposed like the use of dimethyl carbonate for extraction, followed by a purification step with 1-butanol via reflux. After this purification, the overall purity increased from 91.2 ± 0.1% to 98.0 ± 0.1% [51]. Wampfler et al. investigated another possible purification step, particularly experimented for biomedical applications, which implies the filtration through a column filled with activated charcoal (0.5 mL of charcoal per mL of solution to be filtered). The authors stated that endotoxins were almost completely eliminated by this method, removing polymeric impurities with a molecular weight below 10 kDa, as well as the colored impurities [52].

In terms of process development, there are three main steps for industrial PHA production, first the process has to be optimized at laboratory-scale level, and then it is performed in bioreactor and eventually in pilot plant scale with 100–300 L fermenters [53]. After obtaining a globally recognized result at laboratory scale, in the last decades, the industrial PHA market is still gradually increasing, along with the number of independent companies that are investing on PHA production. However, the final result is far from achieved, if we consider, for example, that, in terms of global production capacity, PHA is about 30,000 tons, which is almost ten times less than bio-PE, and almost 20 times less than bio-PET [54]. For successful industrial scale-up PHA production, the influence of oxygen mass transfer and proper agitation are the most important aspects. Therefore, the scale-up strategies need to be based on keeping one of these parameters constant, with respect to the optimized laboratory-scale setup: volumetric oxygen transfer coefficient (K_L_a), volumetric power consumption (P/V), impeller tip speed of agitator (Vs), and mixing time (t_m_) or dissolved oxygen (DO) concentration [55]. To date, worldwide, only a few examples of PHA producers (e.g., Danimer Scientific and Newlight Technologies) have the production capacity to establish collaborations with owners of world-renowned brands in the fields of furniture and food and beverage packaging. This collaboration allows us to boost their economy and lead to a global PHA market growth. 

Concurrently, scientific research and technological innovation are engaged for enhancing PHA production efficiency, by optimizing the biosynthesis mechanisms, valorizing cheap and renewable nutrient substrates, and engineering some new bacterial strains or also mixed microbial cultures (MMCs), which do not require sterile conditions and have a wider metabolic potential than single strain [56].

The great structural variety inside the PHA family is reflected in a wide spectrum of physical properties of PHAs, varying from a stiffer behavior, comparable to polystyrene for PHB, to a more flexible behavior with elongation at break values of PHBV similar to those of polypropylene or even low density polyethylene [57,58]. Generally, PHAs are characterized by a low glass transition temperature, between −50 and 0 °C, and a melting temperature lower than 200 °C [59]. However, probably the most attractive property of PHAs is their biodegradability, which can occur both in aerobic [60] and anaerobic [61] environments, without developing toxic products. The biodegradation of PHAs evolves in three main stages: (1) biodeterioration, which consists in the colonization of the surface, or the bulk of the material, by microorganisms which modify the physical properties of the polymer; (2) biodepolymerization, which is the conversion of polymers into oligomers and monomers induced by enzymes (i.e., PHA depolymerases), secreted by microorganisms, such as bacteria or fungi, which hydrolyze the ester bond of the PHAs; and (3) assimilation, where these low-molecular-weight molecules are metabolized as carbon and energy sources by microorganisms that convert carbon of PHAs into CO_2_, water, and biomass [62,63].

Considering the similarity in mechanical, thermal and barrier properties of PHAs with commodity polymers along with their bio-based origin and biodegradability, this leads to a great interest of PHAs as possible replacements of conventional polymers in different industrial applications [22], such as household or agricultural items manufacturing [64] and packaging [65,66]. However, the higher prices of PHA make them noncompetitive in the current market compared to the fossil-based polymers. In fact, whilst common polyolefins like polyethylene and polypropylene nowadays cost less than 1 €/kg [56], PHAs can range from 2 to 5 €/kg depending on the grade [67]. Their higher prices are mainly due to the cost of carbon sources, substrates and to the low extraction yield at industrial scale [68]. PHAs are largely hydrophobic and soluble in chlorinated hydrocarbons, such as chloroform or dichloromethane. Considering the biomedical applications, the PHA hydrophobic behavior is a suitable property to avoid that the devices undergo a rapid dissolution and a consequent loss of structural properties, once they are implanted in the aqueous body environment. However, it is well-known that wettable scaffolds are conducive to better cellular adhesion, growth and proliferation, due to the ability of maintaining a humid environment and hence promoting fluid exchange between the designed part and the surrounding [69]. In order to tune this hydrophobic behavior, the PHA matrix can be compounded with hydrophilic filler, such as montmorillonite [70], to increase the water affinity of the composites. Two other key properties for PHA medical applications are biocompatibility and biodegradability in physiological environments, which make them suitable for the production of resorbable biomedical devices, which support cellular adhesion, proliferation, and differentiation [49]. A great benefit in biomedicine is the possibility to implant a device that matches the host tissue mechanical property, and hence it decreases stress concentrations at the device–tissue interface. Therefore, the advantage of PHA compared to other polymers clinically used such as poly(lactide-*co*-glycolide) (PLGA), poly(ε-caprolactone) (PCL), poly(glycolic acid) (PGA), or poly(lactic acid) (PLA) is their wide variety of mechanical properties depending on the chemical structure of the monomers. In fact, PLA and PGA have a high Young’s modulus (i.e., 3 and 6 GPa respectively) and a limited elongation at break (i.e., around 2%); differently, PCL has an inferior Young’s modulus (i.e., 0.35 GPa), but a much higher elongation at break (i.e., 400%). These materials are optimal for specific biomedical applications, according to their inherent properties. Due to the possibility of tailoring the Young’s modulus of PHAs, via compounding or synthetic copolymerization, the applicability of this class of biopolymer is potentially much wider and it gives the chance to choose the best grade of copolymer or monomer to mimic the final destination environment [71]. The mechanical properties of human tissue can considerably vary, for example the Young’s modulus for granulation tissue is ~0.2 MPa, for fibrous tissue is ~2 MPa, for articular cartilage is 1–20 MPa, for intervertebral disc is 6–50 MPa, for tendon is 1–3 GPa and for mature bone is ~6 GPa [72,73]. Similarly, the Young’s modulus for PHA family may range from ~600 MPa for some grade of copolymers such as P(3HB-4HB) to ~3 GPa for PHB. It is important to note that also the Young’s modulus of a same copolymer can be tuned by the variation of the molar composition ratio, for example, the P(3HB-4HB) Young’s modulus decreases at the increase of 4HB monomer content [74]. 

Moreover, compared to the abovementioned polymers, PHA has a better interaction with the immune system, due to the unchanged local pH value during its degradation, without toxic or inflammatory reactions [75]. As the other properties, also degradation times for PHAs depend on the chemical structure of the polymer. A previous research study for bioresorbable cardiovascular scaffolds showed that P4HB has a degradation time ranging between two and twelve months. Differently, PGA has an approximate degradation time, starting from six months; PLLA and PCL degradation take longer than two years [76]. 

## 3. Overview on the Main Production Techniques for Biomedical Implants Using PHA

Advances in the biomedical field are not limited to their final applications or the materials used, but they may also concern advancements in the processing techniques of the final implants and devices. Considering the thermoplastic behavior and the solubility in organic solvents of PHA, different approaches have been followed for transforming PHA raw material into architectures with various potential biomedical applications. The first PHA biomedical devices were simple systems with no control on the structure development, and they were obtained by traditional methods, such as (1) solvent casting, (2) salt leaching, (3) thermally induced phase separation (TIPS), (4) non-solvent-induced phase separation (NIPS), (5) emulsification, and (6) electrospinning. Here, the main features of these techniques are reported and summarized.

**Solvent casting** is probably the most common and the simplest technique for polymer film samples production. PHA are dissolved in an organic solvent (e.g., chloroform or dimethyl sulfoxide) at a typical concentration between 2 and 5 wt%; then, the solution is cast into a mold and the solvent is drawn off to obtain a polymer film with a final thickness of about 100 μm [77,78]. An actual problem of this technique is the impossibility of totally controlling the kinetics of the drying process, which could lead to some stress formation into the film structure and to a wrinkled surface. 

**Salt leaching** is a straightforward technique to obtain porous scaffolds, which is a key feature for cell adhesion and proliferation. This process consists in mixing a salt powder, for example, NaCl, with a solution of PHA, and then, after solvent evaporation, leaching out the salt from the structure by soaking the membrane in water [79]. Compared to the solvent cast films, the scaffolds obtained via salt leaching are slightly thicker, varying in a range between 250 and 500 μm [80,81], and with an additional porosity ranging from a few to tens of microns, depending on the size of the salt particles. To avoid using organic solvents, alternatively to the first solvent casting step, a melt molding process is possible. In this case, PHA and salt powders are mixed and poured in a mold, which is first heated above the PHA melting temperature and then cooled down for scaffold solidification. For example, Baek et al. compounded PHBV and hydroxyapatite powder (9:1 *w/w*) with NaCl particles (100–300 μm) at a 1:17 weight ratio and then cast in a mold at 180 °C. The final structure is a porous network with pore sizes ranging from several microns to around 400 μm [82].

**Thermally induced phase separation (TIPS)** is a common alternative approach used in the fabrication of porous PHA scaffolds. The physical principle on which it is based is the changing of the temperature condition of a polymer solution, in order to induce a separation into two distinct phases. First, PHA is dissolved in an organic solvent and then frozen. Next, the solvent is removed by a sublimation process (e.g., freeze-drying), leaving a final porous structure. As an example, You et al. dissolved PHBH in 1,4-dioxane under vigorous agitation at 65 °C, to promote solubilization. The polymer solution was then frozen at −80 °C and lastly freeze-dried for two days. Vacuum drying was applied to completely remove any possible solvent remaining in the scaffolds. Morphology of the scaffolds showed porous structures with pore sizes of approximately 60–100 μm in diameter and 9.3 ± 1.4% in porosity. Moreover, micropores with 5–10 μm diameters were observed interconnected inside the scaffolds, which may help improve intercellular communication [83,84].

**Non-solvent induced phase separation (NIPS)** is another technique used to produce films and thin membranes of PHA. In this case, first PHA is dissolved in an organic solvent and then a phase separation is obtained when this solution enters in contact with a non-solvent, and hence PHA precipitate forming a film. This technique can be used with direct injection in local body sites, and in these cases, it is important to use a non-toxic organic solvent (e.g., dimethyl sulfoxide (DMSO)) for dissolution of PHA, and when this solution comes into contact with aqueous body fluid (a non-solvent for PHA), a PHA membrane is formed, and the polymer solution leads to the precipitation of PHA, which consists in film formation. Dai et al. investigated different non harmful organic solvents: *N*-methyl pyrrolidone (NMP), dimethylacetamide (DMAC), 1,4-dioxane (DIOX), dimethyl sulfoxide (DMSO), and 1,4-butanolide (BL) to be used with PHBH, at 15 wt% concentration, as injectable systems in rats at the intra-abdominal position. The results showed that PHBH films with a porous structure were formed and their surface morphologies depended on the different solvent-exchange rate in the phase separation process involving organic solvents and aqueous liquid. PHBH films prepared from NMP, DMAC, and DMSO showed larger porous structures both on the surface and in the cross-section. Those from DIOX and BL had very low porosity on the surfaces [85].

**Emulsification** is the most prevalent technique to obtain PHA microspheres or nanoparticles, which are further used as drug carriers for pharmacological agents. The derived applications are particularly appropriate for topical therapies at controlled-release rate, to safely achieve the desired therapeutic effects [86]. The oil-in-water emulsion-solvent evaporation method is the standard procedure for PHA nanoparticles fabrication. It consists of mixing an organic phase, PHA polymer dissolved in a solvent, to an aqueous solution with an emulsifier, e.g., poly(vinyl alcohol) (PVA). The organic solvent is then removed by volatilization. Finally, nanoparticles are harvested by centrifugation, washed, and dried. The final dimensions of the nanoparticles are usually between 100 and 200 nm, when ultrasonication is used as mixing step [87,88,89]; differently, if a homogenizer process is used, the dimensions are slightly higher and they vary into a range between 150 and 300 nm [87].

**Electrospinning** is a microfiber production method, and, nowadays, it is the most widely used technique for fabrication of fibrous microporous scaffolds, which simulate the structure of the extracellular matrix. Unlike melt-spinning or wet-spinning, electrospinning does not require a thermal or a chemical coagulation step to produce microfibers. A syringe is filled with a PHA solution and then placed in a high-voltage electric field, usually at 20 kV; thereby, the liquid starts to charge electrically. When the voltage is high enough for the electric repulsion to exceed the surface tension of the droplet at the end of the needle, a thin fluid jet erupts in the direction of the collector, which can be a flat metallic plate or a rotating mandrel. During the travel, the solvent evaporates and the jet dries; hence, electrospun microfibers with a mean diameter of about 500 ± 150 nm [90,91,92] are collected in the form of a microporous film, with a pore size of 1–1.5 μm [92].

**Figure 6:**
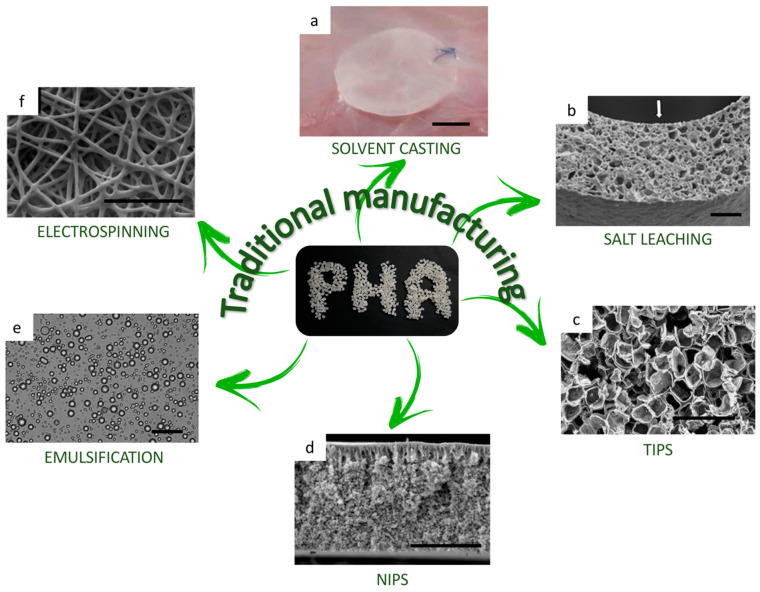
Morphology of PHA scaffolds produced with conventional techniques. (**a**) Visual appearance of a poly(3-hydroxybutyrate-co-3-hydroxyhexanoate), PHBH, film obtained via solvent casting (scale bar = 10 mm; adapted from Reference [93]). (**b**) SEM image of a PHBH conduit cross section with uniform wall porosity obtained via salt leaching; the white arrow indicates the internal side (scale bar = 100 μm; adapted from Reference [81]). (**c**) SEM image of a porous scaffold made of a blend of PHB/PHBH obtained via thermally induced phase separation (TIPS) (scale bar = 500 μm; adapted from Reference [94]). (**d**) SEM image of a PHBV membrane cross-section obtained via non-solvent-induced phase separation (NIPS) (scale bar = 50 μm; adapted from Reference [95]). (**e**) Optical microscopy image of PHBH microspheres prepared via emulsification (scale bar = 50 μm; adapted from Reference [96]). (**f**) SEM image of a porous PHBH film obtained via electrospinning (scale bar = 20 μm; adapted from Reference [92]).

Figure 6 summarizes the above-described conventional processing techniques and graphically represent the final shapes and morphologies of different PHA-based medical devices. 

From the techniques presented so far, we conclude that the sustainability aspect, coming from the production of a bio-based and biodegradable polymer, is undermined by the technological approaches requesting a high amount of harmful organic solvents. Moreover, all these techniques are only suitable for the manufacturing of devices with a very limited 3D structure and, overall, with a maximum thickness of hundreds of microns, which is an evident drawback for an extensive use for biomedical applications.

With the spreading of **additive manufacturing (AM)** techniques, a new light on the modern research scene has been turned on 3D printing for biomedical applications (e.g., tissue engineering, prosthesis, or drug delivery), due to the possibility of tailoring the final design and the manufacturing of complex structures, eliminating the costs and time needed for the construction of molds [97,98]. Three-dimensional printers are commanded by a sequence of instructions, expressed in a computer numerical control programming language (e.g., g-code), to build a three-dimensional object starting from a computer-aided design (CAD) model. Particularly interesting in biomedical applications is the possibility of customizing and elaborating the starting model, in accordance with the morphological structure of the body in which the device is supposed to be implanted, thus achieving optimal compatibility [99,100]. Moreover, with AM approach is possible to tune the mechanical properties of the final device in order to modify the stiffness of the implant to match that of the original tissue, and hence mitigating the problem of stress concentrations. In fact, varying the structure and the design of the 3D-printed device, it is possible to increase the porosity and thereby to decrease of one order of magnitude the Young’s modulus of the implant [73,101,102].

Many different techniques of 3D printing have been invented according to the characteristics of the material processed. For PHA 3D printing, the most applied approach is the one of extrusion-based techniques, in which the biopolymer is either melted or dissolved in a solvent and then extruded through a nozzle and deposited on a printing bed, layer-by-layer. Hereafter, the essential extrusion-based AM techniques used in the production of PHA biomedical applications are discussed and compared to the traditional ones: (1) Direct Ink Writing (DIW), (2) Fused Deposition Modeling (FDM), (3) Selective Laser Sintering (SLS), and (4) Computer Aided Wet-Spinning (CAWS).

**Direct Ink Writing (DIW)** is an extrusion-based 3D-printing technique in which the material is loaded in the form of an ink with rheological properties that allow flowing through the nozzle, as well as supporting its own weight during assembly. In this technique, unlike FDM, the shape retention does not rely on solidification, but rather on shear thinning behavior of the inks. The material is extruded through a thin nozzle, using a computer-controlled robotic deposition system [103]. The final shape of the CAD model is first sliced into layers of height proportional to the nozzle diameter, and it is achieved layer-by-layer. In the production of PHA biomedical devices, the ink is generally obtained by dissolving the biopolymer in a solvent; however, it is also possible to print directly the biopolymer pellets, using a high-temperature print head and thus exploiting the thermoplastic properties of the material. After printing, a final step of cooling or drying occurs, depending if the material underwent a heating process or not.

**Fused Deposition Modeling (FDM)** is the most popular AM technique, due to its straightforwardness and its design freedom. It is a layer-by-layer melt-extrusion approach that consists in heating up a continuous filament of a thermoplastic material above its glass transition temperature (T_g_), and then deposing the extruded material still hot to ensure the adhesion with the underneath layer, already cooled down and hardened. The result is a fully solidified structure whose final design accuracy is guaranteed by a computer control of movements of both printing platform and 3D-printer extruder head [104]. Although FDM can be considered as the most-used 3D-printing technique in a wide range of applications, with different polymeric materials, its utilization for PHA biomedical devices is still extremely limited. Only four scientific research works were published so far, and they evaluate either the applicability as preliminary investigations [105,106,107] or the use of this technique for the production of an external medical aid in the form of a finger cast [108]. 

**Selective Laser Sintering (SLS)** is another AM technique, and it was the first one investigated for production of PHA-based biomedical devices [109]. This approach uses a high-power laser beam to locally sinter the biopolymeric powder bed. This procedure is repeated layer-by-layer, to form a 3D structure with a predesigned architecture, generated by CAD software and transferred to the 3D printer. Due to a suboptimal definition of the sintering process, pore areas of the printed scaffolds are generally reduced, compared to the initial designs. An important influence over this effect depends on the powder layer thickness (PLT) and the scan spacing (SS). Pereira et al. investigated the effect of the variation of these printing parameters over the morphological structure, and it was demonstrated that the increase of SS reduces the size deviation; for example, with a PLT of 0.18 mm and different SS (0.15, 0.20, and 0.25 mm) pores of 0.60 ± 0.04 mm^2^, 0.64 ± 0.04 mm^2^, and 0.68 ± 0.05 mm^2^ were obtained, respectively. Similarly, the increase of PLT also decreased the reduction of pores with respect to the digital model. Printed scaffolds with SS of 0.15 mm showed pore area values of 0.39 ± 0.07 mm^2^, 0.60 ± 0.045 mm^2^, and 0.73 ± 0.07 mm^2^ for PLT of 0.08, 0.18, and 0.28 mm, respectively [110].

**Computer Aided Wet-Spinning (CAWS)** can be considered as an evolution of the wet-spinning technique implemented with a computer control. Wet-spinning consists of extruding from a syringe a PHA solution that precipitates and solidifies in a coagulation bath (e.g., ethanol), due to a non-solvent induced phase separation [111]. The novelty introduced by this technique is the computational control layer-by-layer of the syringe movements, affecting the final shape of the 3D-printed object. This technique allows us to obtain structures with high definition, with a fiber diameter of about 100 ± 20 μm [112,113] and a high porosity, above 80% [112,114]. Due to the non-solvent induced phase separation, this particular technique leads to a multi-scale porous structure in which microporosity, inside the single filaments, is added to a designed macroporous structure. This double scale of porosity has a positive effect on cellular interaction and tissue regeneration [115].

**Figure 7:**
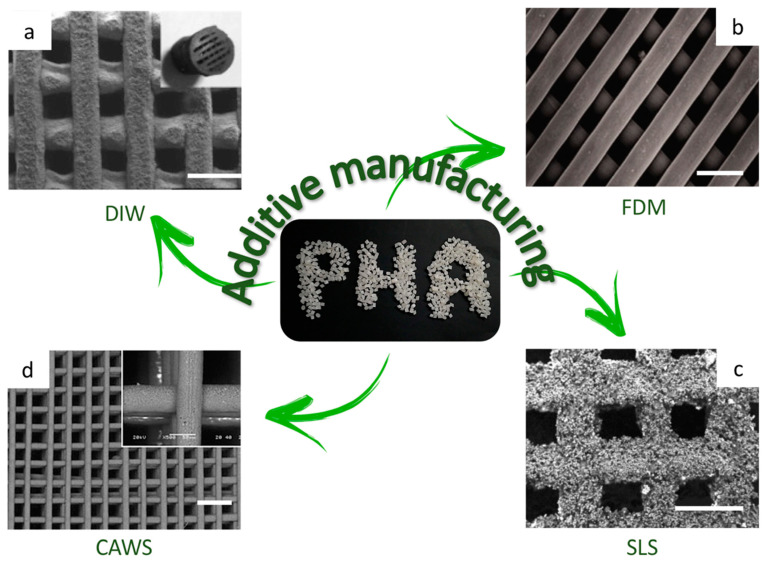
SEM images of PHA scaffolds 3D printed with different AM techniques. (**a**) PHBH scaffolds loaded with anti-tuberculosis drugs 3D printed via Direct Ink Writing (DIW) (scale bar = 500 μm; adapted from Reference [116]). (**b**) PCL/PHBV (50/50) scaffolds 3D printed via Fused Deposition Modeling (FDM) (scale bar = 1 mm; adapted from Reference [107]). (**c**) PHBV scaffolds 3D printed via Selective Laser Sintering (SLS) (scale bar = 500 μm; adapted from Reference [117]). (**d**) Top view of PHBH scaffold 3D printed via Computer Aided Wet-Spinning (CAWS); (insert) detail of the fiber–fiber contact region (scale bar = 500 μm; adapted from Reference [114]).

Figure 7 displays SEM images of scaffolds 3D printed by different AM techniques, showing the final microstructure of the PHA-based medical devices.

All presented techniques used with PHAs for biomedical-device production are summarized and compared in Table 1, with an evaluation of the main advantages and disadvantages of each method.

## 4. Different Biomedical Applications: From Conventional to Innovative Technologies

In the following sections, for a better and clearer understanding for the reader, we decided to use an iterative structure of the paragraphs, dividing every application according to its final utilization: drug delivery, vessel stenting, bone tissue engineering, and cartilage tissue engineering. Then, for each different medical purpose, initially the traditional fabrication techniques of PHA devices are described, highlighting the most important results obtained. Beyond this, the results achieved with AM techniques are illustrated. Particular importance is given to the advancements that AM techniques introduced in the biomedical field, and to the overcoming of some big limitations, which were encountered with traditional techniques. 

### 4.1. Drug Delivery

Drug delivery was the first biomedical application for PHAs that was investigated [124], and in 1983, Korsatko et al. published the first research work for long term medication dosage [125]. Since then, the use of PHAs as drug carriers met a good success in the biomedical field due to their cytocompatibility and their biodegradation properties in different environments. Particularly for drug carriers, the mechanism of PHA extracellular degradation is important since it is strictly related to the amount and the rate of drug released. The basic idea is to degrade the PHA polymer chains into simpler oligomers or monomers and this can occur via lipase-catalyzed chain scission reactions [126] or via PHA depolymerases enzymatic degradation [127]. Both of them substantially hydrolyze carboxyl-ester bonds in alkanol and alkanoic acid, but they differ according to the substrate preference: lipids for lipases and PHA for depolymerases. However, even lipases showed a degradation activity with PHA polymers [128].

The factors that influence the degradation rate of PHA are different and they can be substantially distinguished between environmental factors and intrinsic PHA properties. Generally, we can state that PHA degrades faster in areas with abundance of bacteria, due to an easy colonization of the biopolymer surface by these microorganisms [129]. However, we have to consider also the PHA chemical structure; for example, if we consider PHA with aromatic side chains, not all microorganisms can decompose them [130]. It was found that an increase in anaerobic conditions [131], temperature [132], and humidity [133] can increment, as well, the degradation rate of the PHA, similar to other biodegradable polymers. On the contrary, an inverse correlation was found between the degradation rate and some properties of the PHA, such as the side chain length [134], the molecular weight, and the degree of crystallinity [75]. Therefore, a useful aspect of this biopolymeric family is the possibility of foreseeing a tunable degradation of the final device, according to the particular application. In Table 2, the main correlations between affecting factors and degradation rate are summarized.

The traditional technique that has undoubtedly met the greatest success is the **emulsification** process, which generates nanoparticles that can be loaded with antimicrobial agents or any other drug. One of the first experiments that used the emulsification/solvent diffusion method is dated back to 2008: Yao et al. realized a drug-delivery system that was composed of PHA nanoparticles, phasin (PhaP), and protein ligands. Varying the protein ligands, these systems were tested both in vitro for macrophages hepatocellular carcinoma and in vivo for liver hepatocellular carcinoma. PHAs were suitable for this application, because, due to their hydrophobicity, they had a good affinity with hydrophobic drugs, such as PhaP bound with ligands, which are able to pull the PhaP–PHA nanoparticles to the targeted cells [89].

Xiong et al. demonstrated, for the first time in 2009, the efficiency of employing PHB and PHBH nanoparticles for intracellular controlled drug release via endocytosis by macrophages, which allow the delivery into the cells without receptor mediation. The intracellular drug release was monitored by the amount of change in cells of the retained lipid-soluble colorant, rhodamine B isothiocyanate (RBITC). Both the PHB and PHBH nanoparticles were prepared at two different average sizes of 160 and 250 nm, with a classic **emulsification** procedure, using dichloromethane as organic solvent. It is noteworthy that the drug-loading efficiency decreases with the increase of the PHA nanoparticles dimensions. This study showed that PHA is a class of biopolymer particularly convenient for this application. In fact, it was proved that PHA uptake by macrophages was not harmful for cell viability; moreover, the use of PHA nanoparticles as carriers extends the drug release time. A control sample of free RBITC, not loaded in nanoparticles, was directly added into the culture medium and absorbed by the macrophages in a week. Differently, the use of PHA nanoparticles led to an intracellular sustained drug release period of at least 20 days, meaning an almost threefold increase in drug release time [87].

**Figure 8:**
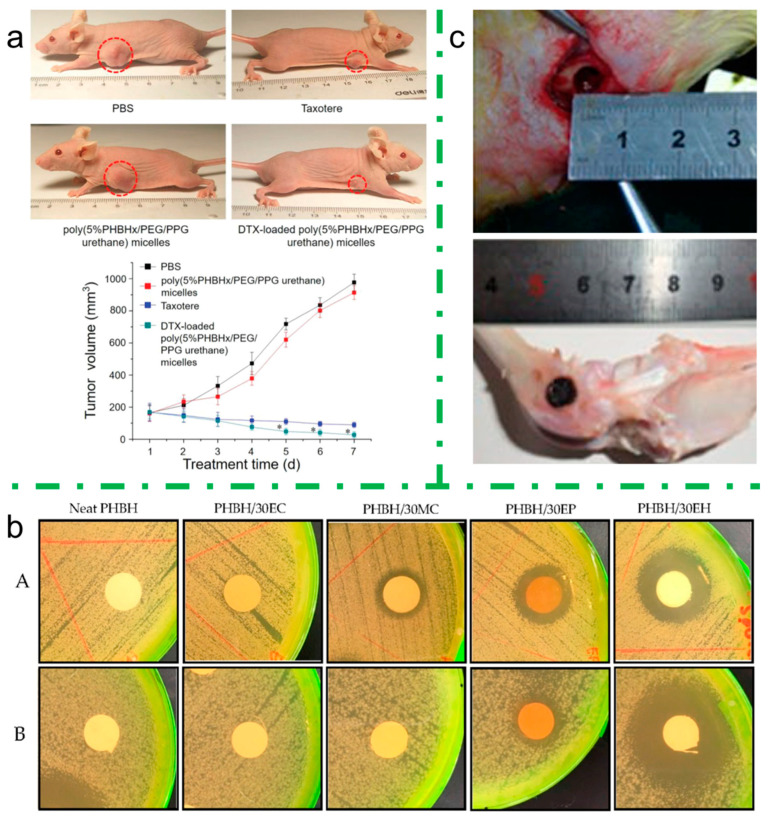
Experimental PHA drug release applications. Panel (**a**) shows in vivo investigation of mice melanoma treatment with PHBH-based polymer micelles loaded with docetaxel (DTX-loaded poly(5%PHBHx/PEG/PPG urethane) and different control groups: Phosphate Buffered Saline (PBS), a commercial docetaxel treatment (Taxotere), and unloaded PHBH-based polymer micelles (poly(5%PHBHx/PEG/PPG urethane). Visual appearance of subcutaneous tumor sizes and tumor volume measurements, within treatment time, are displayed at the top and bottom of the panel, respectively (adapted from Reference [135]). Panel (**b**) represents the inhibition zones of neat PHBH and PHBH composite electrospun nanofibers with centella (30EC) and (30MC), propolis (30EP), and hinokitiol (30EH) on Gram-positive bacteria (*S. aureus*) (A) and Gram-negative bacteria (*E. coli*) (B) (adapted from Reference [91]). Panel (**c**) shows two photographs of a cylindrical scaffold (D × H = 6 × 8 mm^2^) 3D printed via DIW and implanted in a rabbit’s femur for post-surgical treatment of osteoarticular tuberculosis (adapted from Reference [137]).

More recently, Luo et al. used the **emulsification** technique to produce some PHBH-based polymer micelles loaded with docetaxel (DTX) for melanoma treatment. The PHBH-based system is particularly useful to encapsulate DTX, because it avoids using nonionic surfactants that are currently employed for marketed DTX product and that are reported to cause hemolysis, hypersensitivity reactions, or neuro-toxicity. Interestingly, this micelle formulation shows a drug loading efficiency higher than 90%, it improves DTX solubility in aqueous medium and it reduced hemolysis for better blood compatibility. In vivo tests were run by subcutaneous inoculation of a solid tumor, A375 cells, in a mouse and then applying and comparing a control test with PBS (Phosphate Buffered Saline), a marketed DTX treatment and a DTX-loaded PHBH-based micelle treatment. After a week, the results showed an expected increase of about 450% in final tumor volume for the control group, whereas with commercial DTX and experimental micelle the melanoma underwent a volume reduction of 50% and 80%, respectively. Therefore, the results, shown in Figure 8a, demonstrated not only a better blood compatibility but also a better inhibitory ability of the DTX-loaded PHBH-based micelle, compared to a commercial DTX treatment [135].

Rebia et al. produced a fully natural nanofiber composite via **electrospinning** that can mimic the native extracellular matrix (ECM), and therefore increase the compatibility with the host body. The researchers loaded a PHBH matrix with natural antibacterial reagents (*Centella*, propolis, and hinokitiol) to produce antibacterial wound dressings. The obtained structures have a thickness varying from 50 to 140 μm, and they can withstand only moderate mechanical stresses. The in vitro antibacterial activity was evaluated by using the inhibition zone method both for Gram-positive bacteria, tested with *S. aureus*, as well as for Gram-negative bacteria, tested with *E. coli*. The results with propolis and hinokitiol loading gave promising outcomes (Figure 8b) [91].

Traditional techniques are positively used to fabricate drug nanocarriers, but the biggest limitation is that the obtained devices have a very low versatility in the design structures, which are thin membranes in the case of electrospinning or nanospheres obtained by emulsification. Therefore, the introduction in this application field of AM permitted to obtain complex architectures, extended in all three dimensions, which could operate not only as drug carriers but also as structural support, in the target site.

Duan et al. was one of the first researchers that investigated the 3D printability of PHA for biomedical application. Starting from a micropowder, obtained by double emulsion solvent evaporation method, the researchers decided to further use it, not as a simple drug carrier, but as a powder bed for **SLS** technique. First, a calcium phosphate (Ca-P)/PHBV composite powder loaded with bovine serum albumin (BSA) was prepared, and then the scaffolds (L × W × H = 8 × 8 × 15.5 mm^3^) were designed and 3D printed [136].

Li et al. and Zu et al. suggested an interesting application for a mesoporous bioactive glass (MBG) and PHBV composite, 3D printed via **DIW** starting from a polymer ink dissolved in chloroform and dimethyl sulfoxide. The final goal of this application is meant for post-surgical treatment of osteoarticular tuberculosis, and specifically the 3D-printed scaffolds can be implanted in the surgical defect, combining the osseous regeneration effect with the release of an antituberculotic drug, such as isoniazid or rifampin. The studies investigated in vitro drug release and cellular proliferation, and in vivo surgical procedure was run, implanting the 3D-printed cylindrical scaffolds (D × H = 6 × 8 mm^2^) into the femur of different rabbits, represented in Figure 8c. Besides the osteogenetic feature of this material, another attractive property is the slower and controlled release of antituberculotic drug, up to three months, lengthening the healing period and reducing systemic side effects [116,137].

Wu et al. investigated the possibility of 3D printing a clinical device via **FDM**, which could also have an antibacterial activity. They melt-compounded a maleic anhydride grafted PHA (PHA-g-MA) with multi-walled carbon nanotubes (MWCNTs) for the production of a FDM filament, which can be further used to 3D-print different geometries according to the final application. Only a preliminary study of the antimicrobial assay was tested with the inhibition zone method both for Gram-positive bacteria, tested with *S. aureus*, and for Gram-negative bacteria, tested with *E. coli*. Generally, the tested samples demonstrated a higher inhibition zone for *E. coli* rather than *S. aureus*; however, for both class of bacteria, the results showed an increase in antibacterial performance following an increase in MWCNTs content [106]. 

### 4.2. Vessel Stenting

One of the most recent developing field of PHA application is the stent vessel production, since biodegradable stents can provide mechanical support while it is needed, for example, for obstructive cardiovascular disease treatments, and then degrade, leaving behind only the healed natural vessel, without any foreign objects in the body. 

For vascular application, the most important biological property of PHA to investigate is the hemocompatibility, for example with an erythrocyte contact hemolysis assay. The easiest way to do that was to prepare **solvent cast** films. Qu et al. fabricated samples of PHB, PHBV, and PHBH. Comparing all the films, the best results were obtained with PHBH films, which showed a two-fold reduced hemolytic activity and also a lower number of bound blood platelets, after a 120-min exposure to platelet-rich plasma [138]. Zhang et al. tried to improve other important properties of PHBH, in order to enhance the applicability of this PHA in vascular engineering. Particularly, they blended PHBH and poly(propylene carbonate) (PPC) to obtain a higher flexibility, evidenced by an increase in elongation at break [118].

**Figure 9:**
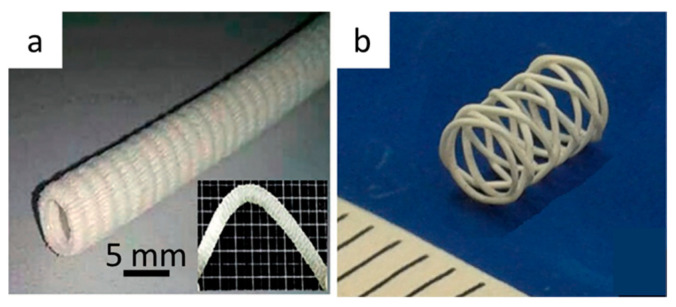
Experimental PHA applications for vessel stenting. (**a**) Macro morphology of corrugated tubular PHBH scaffold obtained via electrospinning (adapted from Reference [139]). (**b**) Representative photograph of a stent 3D printed via CAWS for small-caliber blood vessels (measure unit = 1 mm; adapted from Reference [123]).

The former studies were fundamental to characterize and to state the possible use of this class of polyester for vascular engineering applications. However, there was a big technological issue with this traditional technique, because solvent casting is not suitable for the production of final devices with complex and 3D structures, which are meant to be implanted in human blood vessels. Gao et al. suggested the use of **electrospinning** to fabricate two kinds of PHBH vascular grafts, including straight and corrugated structures with 6 mm inner diameters. These devices have been tested mechanically, to undergo radial compression and circumferential tensile stresses, as well as for suture retention strength and radial compliance. Moreover, the biocompatibility was evaluated in vitro with hemolytic and cytotoxicity tests. The results obtained in this study demonstrated good application value in the field of stent vessel engineering, even comparing the final properties of the experimental grafts with those of commercial ones [139]. Electrospinning is a well-known technique for production of microporous films, but the production of devices with an actual 3D structure is time-consuming. For example, in this study, the realization time of a vascular graft with a thickness of 200 μm took 6 h. Figure 9a shows the final macroscopic aspect of such electrospun PHBH vascular grafts.

Even if the volume of research is still limited, the innovation that AM introduced in this subject of study is noteworthy. Balogová et al. carried out a preliminary study for production of urethra replacement via AM. They prepared a prototype via **DIW** a PLA/PHB tubular structure with the same length and thickness of the aforementioned vascular grafts, which only took 10 min. Compared to the previous research, the use of AM allowed a 36-fold reduction in production time, which is an evident advantage for technological applications. In this first research study, the authors focused on the technological aspect of the production, and they investigated only geometrical and viscoelastic properties of 3D-printed samples, such as the shape retention over time and the deviation from designed sizes. It is possible to state that DIW had sufficient precision to produce tubular samples usable as a replacement for urethra; further mechanical and biological characterizations have to be done to further validate the in vivo implantation [140].

Puppi et al. realized via **CAWS** some PHBH stents for small-caliber blood vessels, and one example is shown in Figure 9b. The developed stents sustained proliferation of human umbilical vein endothelial cells in vitro, and they showed encouraging low levels in terms of thrombogenicity when in contact with human blood. Besides the advance in medical application, this study is also technologically interesting because it widened the field of application of the CAWS technique. It introduced a novel approach that allows the construction of 3D tubular structures by winding the coagulating wet-spun biopolymer fiber around a rotating mandrel with a predefined pattern. The biopolymer solution is extruded through a needle directly above a rotating mandrel immersed in a non-solvent bath of ethanol; the movement of the needle and the mandrel rotational velocity were controlled by an experimental computer-controlled system. The presented technique showed a great versatility in the customization of stent fabrication [141].

### 4.3. Tissue Engineering

A challenging frontier of modern medicine is the repairing of damaged tissue of the human body, and it is called regenerative medicine. The main goal of this particular application field is to promote and enhance the formation of new viable tissues by biochemical and cellular processes. A key feature is represented by the positive effects of biocompatible materials and the innovations of technologies that can enhance the fabrication of devices able to simulate the original body environment. In order to achieve this, a connection among different disciplines (biomedicine, material science, and engineering) has to be done, and for this reason, a new interdisciplinary research field was created, i.e., tissue engineering. Due to the good cytocompatibility and to the tunable mechanical properties and degradation rate, PHA demonstrated to be suitable for both hard tissues, i.e., bone and cartilage, and soft tissues [142], nerve, tendon, bone marrow, or vascular applications. In tissue engineering the device customization is a great advantage; therefore, we can state that this area is the most promising and with the highest potential for biomedical 3D printing.

#### 4.3.1. Bone Tissue Engineering

**Figure 10:**
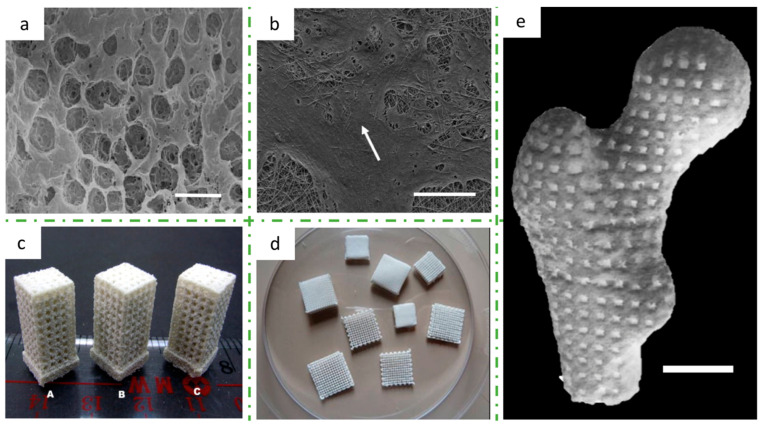
Experimental PHAs applications for bone tissue engineering. (**a**) SEM images of a porous PHBV scaffold obtained by salt leaching (scale bar = 10 μm; adapted from Reference [144]). (**b**) SEM micrographs of PHBH/silk fibroin (1:1) electrospun films after 14 days of human-umbilical-cord-derived mesenchymal stem cells culture. The white arrow indicates the cells homogeneously distributed on the microporous film (scale bar = 150 μm; adapted from Reference [92]). (**c**) Visual appearance of Ca-P/PHBV scaffolds loaded with BSA and 3D printed via SLS, using different sintering parameters: (A) laser power = 12.5 W and scan spacing = 0.1 mm; (B) laser power = 15 W and scan spacing = 0.1 mm; (C) laser power = 15 W and scan spacing = 0.15 mm (adapted from Reference [136]). (**d**) Visual appearance of PHBH scaffolds 3D printed by CAWS (adapted from Reference [114]). (**e**) Calcium phosphate (Ca–P)/PHBV nanocomposite 3D printed via SLS for the fabrication of a proximal femoral condyle scaffold (scale bar = 1 cm; adapted from Reference [147]).

One of the first in vitro research studies of biocompatibility of PHBH was conducted by Yang et al., and they demonstrated that bone marrow stromal cells can attach, proliferate, and differentiate into osteoblasts on PHBH films, obtained by **solvent casting** [143]. Wang et al. used the **salt-leaching** technique to obtain porous scaffolds, in order to demonstrate an increased attachment and proliferation of bone marrow cells, as well as an earlier osteogenesis, onto a rough surface. The optimal pore size detected is about 3 μm in diameter. In this study, PHBH scaffolds (Figure 10a) showed better performance for osteoblast proliferation rather than PHB and PLA scaffolds [144]. The same authors investigated also the compounding of PHB and PHBH with hydroxyapatite (HAP), and they found that the mechanical properties (compressive elastic modulus and maximum stress) and the osteoblast response improved for the PHB matrix and decreased for the PHBH blend [145]. More recently, Wu et al. studied how to enhance the cell compatibility of the PHA matrix varying the surface morphology of the solvent cast film by compounding the PHBH with carbon nanotubes (CNTs), which resulted in a higher surface roughness and an electrical conductivity. The proliferation of human mesenchymal stem cells (hMSCs) were demonstrated to be outstanding when nanocomposite films contained 1 wt% CNTs, compared with that on pristine PHBH [77]. 

Assuming that porosity is an increasing factor of cellular proliferation, Xi et al. investigated the possibility of controlling it and they identified **TIPS** as a straightforward technique that allows the regulation of the scaffold pore diameters by varying the quenching temperature and time. The researchers obtained a series of interconnected highly porous scaffolds with pore sizes ranging from 30 to 150 μm. They demonstrated that the pore diameter decreases with decreasing quenching temperature and consequently also the overall porosity of the scaffold [146].

Ang et al. successfully fabricated **electrospun** films made of PHBH compounded with silk fibroin (SF), and these devices were able to support the human umbilical cord-derived mesenchymal stem cells proliferation and differentiation into the osteogenic lineage. The obtained electrospun films are in the form of a porous matrix with randomly distributed fibers, with an average diameter in the range of 600 and 980 nm. The mean pore diameter of the electrospun films ranged from 1 to 1.5 μm. Silk fibroin demonstrated an enhancing effect on the proliferation and osteogenic differentiation of stem cells, compared to the pristine PHBH. In Figure 10b, the spread of the cells over the electrospun membrane is shown [92].

Even if the techniques described so far have been instrumental in starting to investigate the use of PHA for bone regeneration, traditional scaffolds have some big limitations, such as very little thickness (i.e., hundreds of microns) and no real direct control over porosity, nor over the dislocation and size of the pores. These aspects have been positively overcome with the use of AM, which has widened the range of application. Three-dimensional printing allows us to build geometries with customized and controlled designs, including the internal pattern, and with a development even in height of several centimeters.

**SLS** was the first AM technique used to fabricate PHA 3D scaffolds. Pereira et al. realized a tetragonal structure squared base (13 × 13 mm^2^) and 26 mm high, with a designed porosity of 1 mm^2^ area. PHB scaffolds were 3D printed with different properties, due to the change in the values of the scan spacing (SS) and powder layer thickness (PLT). The results showed that a decrease of the values of PLT or SS involved an increase in the compressive mechanical properties of scaffolds, such as ultimate compressive strength and compressive modulus [110].

Duan et al. studied a system that provided a biomimetic environment for cell attachment, proliferation and differentiation, based on a composite of PHBV compounded with calcium phosphate (Ca-P) nanoparticles, which was proved to be an osteoconductive component. The researchers carried out a study aimed to optimize the **SLS** 3D-printing parameters, i.e., laser power, scan spacing, and layer thickness, according to the final resolution and mechanical properties of a tetragonal porous scaffold (L × W × H = 8 × 8 × 15.5 mm^3^), of which three examples are shown in Figure 10c [122]. The final nanocomposite revealed to have not only positive mechanical properties but also good cytocompatibility, tested with a human osteoblast-like cell line [117]. To prove the possibility of using the SLS technique for real medical applications, a human proximal femoral condyle model was obtained from computer tomography scans and then 3D printed into a porous scaffold model with a pore size of 2 mm; an image of this medical prosthesis is shown in Figure 10e [147]. 

In 2013, **DIW** was investigated by Yang et al. for the first time, among all extrusion-based AM approaches, as a possible technique for PHA bone scaffolds production. Yang et al. fabricated composite scaffolds made of PHBH and mesoporous bioactive glass (MBG) through a combination of 3D printing and surface doping. The MBG coating was found to improve surface hydrophilicity and bioactivity, as well as provide a better environment for human mesenchymal stem cells viability, proliferation, and osteogenic differentiation [148]. Based on the promising results of in vitro biological characterization of the nanocomposite, the research was further carried out by Zhao et al., who selected MBG/PHBH composite scaffolds 3D printed via DIW for in vivo evaluation of osteogenic capability. The scaffolds stimulated bone regeneration in rat calvarial defects within eight weeks [149].

Li et al. studied a real application case of interest for a PHBH and MBG drug-loaded scaffold for osteoarticular tuberculosis. After surgery, it is necessary to fill the surgical defect with an implant, which can combine the effects of osseous regeneration and antitubercular drug (e.g., isoniazid and rifampin) local delivery to treat the area affected by the disease and to avoid internal infections. The researchers 3D-printed, via **DIW**, a cylindrical porous scaffold with a height of 8 mm, a diameter of 6 mm, and an area of each pore of 0.25 mm^2^. The AM technique was particularly useful in this application, to realize a customized device that could perfectly fit to the size of the hole surgically drilled into the treated bone. The structure was tested both for in vitro compatibility and in vivo implantation in a rabbit femur defect model (Figure 8c). Microtomography evaluations and histology results indicated part degradation of the composite scaffolds and new bone growth in the cavity [116,137].

Mota et al. explored another innovative 3D-printing technique, which was used for the first time with PHA, the **CAWS**. In this study, PHBH 3D-printed scaffolds with different pore sizes and internal architectures were fabricated layer-by-layer, and the processing parameters were investigated for optimization of mechanical compressive properties and biological evaluation. The scaffolds showed a porosity of 79–88%, an extruded filament diameter of 47–76 μm, and a pore size of 123–789 μm; hence, this AM technique allowed the fabrication of scaffolds with a high resolution and a good control over scaffold external shape and internal pattern. The PHBH scaffolds demonstrated also promising results in terms of cell differentiation towards an osteoblast phenotype [114]. Puppi et al. carried on the investigation on PHBH 3D printing for bone scaffold regeneration via CAWS with a pristine PHBH matrix (Figure 10d) [115] and with a PHBH/PCL blend composition [113]. All results showed a promising applicability for in vivo studies and implantations. Recently, they published a work where they used a ternary mixture of PHBH/chloroform/ethanol to prepare the polymeric ink to be used in the 3D printer. With this method, they suggested a more sustainable CAWS process for PHBH scaffolds production, which reduces the employment of halogenated solvent by replacing with ethanol up to 40 *v*/*v*% of the chloroform employed. Besides thus, they evaluated the effect of varying the solvent/non-solvent ratio on structural morphology, such as macro- and microporosity, on tensile properties and on in vitro preosteoblast cells proliferation [112].

#### 4.3.2. Cartilage Tissue Engineering

Differently from bone regeneration, cartilage structure cannot be self-recreated and an excessive wear of this tissue can lead to a cartilage loss and to osteoarthritis problems. Currently, the most common treatments involve only the use of painkillers or surgeries, such as microfracture, osteochondral transfer or autologous chondrocyte implantation. However, these treatments present no actual restoration of cartilage tissue and, in general, an unsatisfactory average long-term result [150]. In the last decade, a new approach for cartilage repair was suggested, and it consists in the use of engineered scaffolds able to support the growth of chondrocytes. However, still further research is required to develop suitable scaffolds, because the neo-generated tissue is often fibrocartilage, which is mechanically inferior and less durable than the one found in healthy articular joints [151]. Since the beginning of the investigation, a particular interest was attributed to PHA as interesting material for the recreation of a favorable environment for the growth of chondrocytes from stem cells. The first works focused on the interaction of chondrocytes with polymer matrices. Deng et al. blended PHBH and PHB and then porous scaffolds were fabricated by the **salt-leaching** method. In order to evaluate the compatibility with this material and the production of extracellular matrix, the chondrocyte cell lines were isolated from rabbit articular cartilage, seeded on the scaffolds and incubated over 28 days [80,152]. Following research explored the best ratios between different component polymers, which could positively combine mechanical properties and biological compatibility. Considering collagen II as a differentiation marker of chondrocytes maturation, blended scaffolds of PHB and PHBH (ratio 1:2) gave the best results, compared with other ratios of PHB/PHBH or even with PLA [153].

The **TIPS** technique was used as another simple approach to fabricate PHB/PHBH porous scaffold upon which human adipose-derived stem cells (hASCs) were seeded to produce neocartilage, subsequent to a chondrogenic differentiation in vitro process. After 14 days of in vitro culture, the differentiated cells grown on the PHB/PHBH scaffold were implanted into the subcutaneous layer nude mice and after 24 weeks, the appearance of a new cartilage-like tissue could be observed [94]. To develop a higher and more homogeneous cell proliferations over the PHBH scaffolds, You et al. experimented a biological coating of the biopolymer scaffolds with PHA granule binding protein (PhaP) fused with RGD peptide (PhaP-RGD coating). Human bone marrow mesenchymal stem cells (hBMSCs) were inoculated in the scaffolds and the findings showed that the proposed PhaP-RGD coating led to a more homogeneous spread of cells, and to a better cell adhesion, proliferation and chondrogenic differentiation [84].

**Figure 11:**
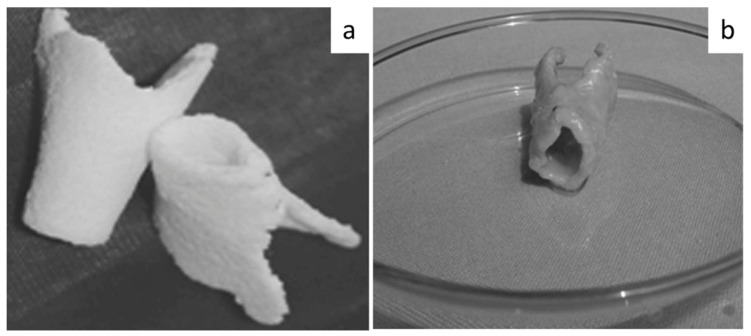
Experimental PHAs applications for cartilage tissue engineering. (**a**) Representation of a PHBH medical device prepared by solvent casting, compression molding, and particulate filtering, with a final hollow semi-flared shape, which intends to mimic the laryngeal cartilage morphology (adapted from Reference [154]). (**b**) Photograph of the laryngeal cartilage PHBH specimen with chondrocytes inoculated, 18 weeks after implantation (adapted from Reference [154]).

All mentioned research studies provided a strong and valid basis to start investigating the applicability of PHA matrices for cartilage tissue engineering, but a big limitation was represented by the geometrical constraint in the final shapes of the devices obtained by TIPS or salt leaching. Starting from a real case study, Sun et al. analyzed a possible and new route to build and replace a damaged laryngeal cartilage. The noteworthy innovation of this work was the construction of a hollow, semi-flared geometry prepared by a **combination of solvent casting, compression molding** in a polytetrafluorethylene form, **and salt-leaching** methods. The morphology of the implant was shaped according to the anatomy of an adult laryngeal cartilage, as can been seen in Figure 11a. First, chondrocytes were inoculated onto the PHBH scaffold, and after one week of in vitro culture, an in vivo implantation was performed and the results showed that cartilage formed six weeks after the surgery (Figure 11b) [154].

The former work had the great advantage to allow the construction of a complex-shaped device; nevertheless, the experimental procedure for the scaffold fabrication is long and expensive, since it involves using a plastic mold, which should be, every time, customized according to the final implant. Moreover, organic solvent and long times of evaporation need to be estimated. With AM, these limitations could be easily overcome, because starting from a different CAD model, the need for the mold would be completely eliminated. Moreover, 3D printing would allow the fabrication of personalized and complex structures, which could encourage cellular growth in preferential directions or which could have architectures that optimize the contact and the stress transmission between bone and cartilage, for example, in the case of articular cartilage. To the authors’ knowledge, there is only one recent work dealing with 3D printing of PHA scaffolds for cartilage tissue engineering. De Pascale et al. assessed the properties of collagen I hydrogel 3D scaffolds, strengthened with solvent cast and 3D-printed PHA polymer. The addition of solvent cast and 3D-printed scaffolds increased the mechanical resistance of the structures when compared to the collagen matrix only. Once again, the use of AM technique was an advantage related to traditional techniques, because regarding the compressive stress that the device could undergo, 3D-printed scaffolds showed the highest stiffness compared to the collagen and solvent cast polymer samples [155].

## 5. Future Perspective

The introduction of PHA and AM in the biomedical field has boosted the advancements of innovative solutions for problems that were so far totally or partially unresolved. The main reasons for this success was certainly due to the high level of customization brought by AM and by the possibility of tailoring the final mechanical properties of 3D-printed materials, in order to mimic the tissue environment. Besides, also the tunable and interesting properties of PHAs played a central role, for example the wide processing and application versatility, the biological origin, the biocompatibility and the biodegradability. Among AM techniques, FDM owns some well-known advantages, namely its simplicity, rapidity, and ecological sustainability; in fact, it does not require the use of any organic solvent. However, FDM used with PHA for biomedical application is still limited; however, according to the abovementioned properties and advantages of PHA and FDM, we believe that its use will be increasingly investigated and the number of 3D-printed devices by FDM will grow significantly in the next years.

In the field of PHA 3D-printed medical devices, the most promising results were obtained with non-toxic and safely resorbable scaffolds containing living cells that were used for hard tissue regeneration, bone and cartilage particularly. However, no studies were carried on the production of more complex-shaped devices like prosthesis or surgical implants, because these are generally 3D printed with synthetic biopolymer, such as PCL.

**Figure 12:**
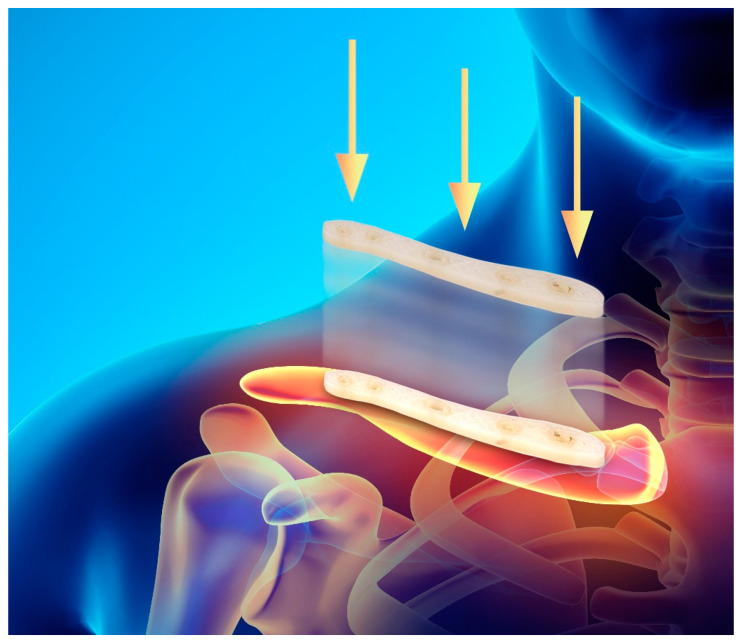
Graphical representation of a PHBH clavicle plate 3D printed by FDM and its final surgical application for bone regeneration (adapted from istock.com/yodiyim, accessed on 21 September 2020).

The production of synthetic polymer requires the use of chemical solvents, different catalysts (e.g., metal-based, organic, or even enzymatic systems) and also reaction conditions that are particularly energy consuming [156]. If compared to a bacterial synthesis of PHAs, it is quite evident the inconvenience in terms of ecological sustainability. As an indication of possible future developments, in Figure 12 a PHBH clavicle plate 3D printed by FDM is shown, which could be used to treat a broken fracture. Especially due to the resorbability, to the biocompatibility and to the osteogenesis induction of PHAs, this class of material allows us to think of a future medicine, where all components are bio-based, perfectly compatible with human body and devices can be harmlessly reabsorbed by our organism, when they are not needed anymore.

In conclusion, we can foresee a quick and important development in this research field and we think that the next frontier and challenge in biomedical application of PHA could be the 3D printing by FDM of entire prosthesis, or complex surgical implants, which can replace the materials used until now, and which will notably improve the biomedical knowledge and technological state-of-the-art.

## Figures and Tables

**Table 1:** Outline and comparison of the traditional and additive manufacturing (AM) techniques used to produce medical devices from PHA.

	Technique	Final Device Shape	Advantage	Disadvantage	Reference
Traditional Techniques	Solvent Casting	film/membrane	+ Easiness and low cost	− Limited to 2D structure − Use of organic solvent − No control on stress formation during drying process	[118]
	Salt Leaching	scaffold	+ Easiness and low cost + Indirect control on pore size	− Small thickness − No customization	[119]
	TIPS	scaffold	+ High porosity	− Use of organic solvent − No control on porosity structure − Energy consuming	[120]
	NIPS	film/membrane	+ Easiness and low cost	− Use of organic solvent − No control on final geometry	[120]
	Emulsification	microspheres	+ High surface/volume ratio	− Limited design freedom − No significant 3D development	[121]
	Electrospinning	microporous film	+ Easiness and low cost	− No significant 3D development (thin films) − Dependent on environmental conditions (humidity)	[46,121]
AM techniques	DIW	scaffold	+ Rapidity of processing + Complex geometries + High resolution (low layer height)	− Use of organic solvent − Solvent evaporation (post printing)	[103]
	FDM	scaffold	+ Easiness and low cost + Fast printing speed + Roughness of surface (cell attachment) + Solvent-free process + Complex geometries	− Lower resolution − High temperature processing	[104,105,108]
	SLS	scaffold	+ No need to support material	− Big minimal amount of material − High and not controlled porosity due to not perfect sintering	[122]
	CAWS	scaffold	+ Rapidity of processing + High resolution (low layer height)	− Use of organic solvent	[123]

**Table 2:** Main correlations between the degradation rate and affecting factor of degradation. The ↑ symbol indicates an increase; the symbol ↓ indicates a decrease.

	Factor	Degradation Rate	Reference
Environmental factor	↑ microbial population	↑	[129]
	↑ anaerobic condition	↑	[131]
	↑ temperature	↑	[132]
	↑ humidity	↑	[133]
PHA Properties	↑ side chain length	↓	[134]
	↑ degree of crystallinity	↓	[75]
	↑ molecular weight	↓	[75]
